# Immediate Euglycemic Diabetic Ketoacidosis After Gastric Bypass in a Patient with Type II Diabetes

**DOI:** 10.1007/s11695-021-05263-7

**Published:** 2021-02-10

**Authors:** Yu-Ting Lo, Kai-Hua Chen, Po-Chih Chang

**Affiliations:** 1 Department of Surgery, Kaohsiung Medical University Hospital/Kaohsiung Medical University, Kaohsiung City, Taiwan; 2 Division of Thoracic Surgery, Department of Surgery, Kaohsiung Medical University Hospital/Kaohsiung Medical University, Kaohsiung City, Taiwan; 3 Weight Management Center, Kaohsiung Medical University Hospital/Kaohsiung Medical University, No. 100, Tzyou 1st Road, Kaohsiung City, 80756 Taiwan; 4 Ph. D. Program in Biomedical Engineering, College of Medicine, Kaohsiung Medical University, Kaohsiung City, Taiwan; 5 Department of Sports Medicine, College of Medicine, Kaohsiung Medical University, Kaohsiung City, Taiwan

**Keywords:** Bariatric surgery, Diabetic ketoacidosis, Euglycemic diabetic ketoacidosis, Type II diabetes mellitus

Bariatric surgery has been proven to be an effective solution for morbid obesity with type 2 diabetes mellitus (T2DM), and such patients usually receive oral hypoglycemia agents or insulin for blood sugar control prior to surgery [1]. Though the occurrence of euglycemic diabetic ketoacidosis (DKA) is not clinically infrequent especially for those on treatment with sodium-glucose cotransporter 2 (SGLT2) inhibitor, reports of DKA after bariatric surgery are limited and most of the available reports discussed type I diabetes [1–3].

Herein, we report a T2DM patient (without SGLT2 inhibitor use) who developed euglycemic DKA soon after Roux-en-Y gastric bypass (RYGB). Moreover, the postoperative course was complicated with an attack of chronic gouty arthritis and acute exacerbation of chronic kidney disease, but eventually the patient made a full recovery.

A 52-year-old, morbidly obese male patient (body weight 110.3 kg; body mass index 37.7 kg/m^2^), with newly diagnosed T2DM (glycated hemoglobin 9.5%; homeostatic model assessment of insulin resistance 8.2) and poly-morbidities (hypertension, dyslipidemia, hyperuricemia with left ankle tophi, chronic kidney disease, and non-alcoholic fatty liver disease), was referred to our hospital for weight loss surgery. For blood sugar control, only metformin was prescribed (500 mg thrice a day) instead of a SGLT2 inhibitor. The patient had also taken diclofenac occasionally to relieve pain from chronic gouty arthritis over the bilateral feet. Laparoscopic RYGB was uneventfully performed on the 2nd day after admission with an estimated blood loss of 30 mL. The operation time was 210 min, and the intra-abdominal pressure of the pneumoperitoneum was up to 15–17 mmHg during the surgery.

**Fig. 1:**
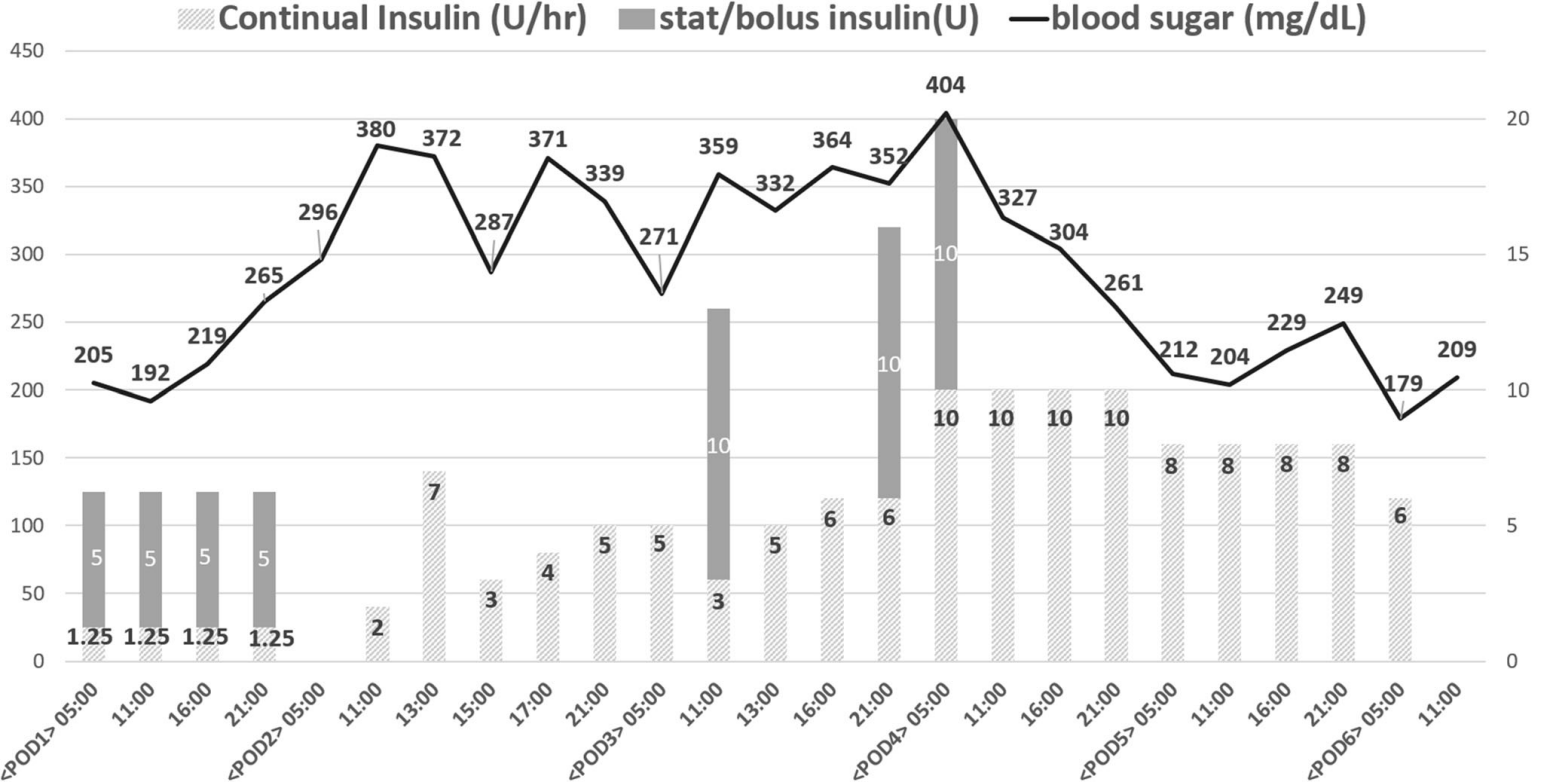
Timeline of blood sugar level and regular insulin dose within the early postoperative stage. We infused regular insulin with a pump from postoperative day 2 to 6

On postoperative day 1, the patient started sipping water without obvious discomfort, and regular insulin was continuously infused at a rate of 1.25 U/h (blood sugar level was between 192 and 205 mg/dL) (Fig. 1). However, a spiking fever (39.3°C) occurred in the evening with a significant decrease in urine amount (from 3010 to 480 mL/day). Meanwhile, the patient also presented with tachypnea (26 breaths per minute) and tachycardia (123 beats per minute). No peritoneal sign or chest tightness was reported. The laboratory studies showed leukocytosis, elevated level of procalcitonin (3.4 ng/mL), and deteriorated renal function (creatinine 3.99 mg/L) (Table 1). Moreover, the blood gas analysis demonstrated metabolic acidosis with respiratory compensation (Table 1). The blood sugar still remained at a marginally higher level (219 mg/dL). Upon arriving at the intensive care unit, hypotension developed (72/49 mmHg) and fluid resuscitation, vasopressor, and empirical antibiotics (imipenem + cilastatin) were administered for possible occult intra-abdominal infection.

**Table 1:** Laboratory studies from postoperative day 1 to day 5. *pH* potential of hydrogen, *pCO2* partial pressure of carbon dioxide, *BUN* blood urea nitrogen, *eGFR* estimated glomerular filtration rate

	POD1		POD2		POD3	POD4	POD5
Serum glucose level (mg/dL)	205	219	296	371	359	404	212
pH		7.322	7.304	7.386	7.4	7.38	7.415
pCO2 (mmHg)		32.6	28.6	35.9	43	42	45.5
Bicarbonate (mmol/L)		16.5	13.9	21.1	26	24.3	28.5
Anion gap				19			10
Osmol (mOsm/kg)				317			
Serum ketones (mmol/L)			6.1	4.3	0.7		
White blood cell (/uL)		15,070	20,110		7660	4880	2900
Hemoglobin (g/dL)		13.8	12.5		34.9	35.3	34.7
Platelet (/uL)		182,000	201,000		134,000	119,000	135,000
Procalcitonin (ng/mL)		3.4			13.27	19.5	37.04
C-reactive protein (mg/L)		193.02					291.41
Lactate (mmol/L)			1.7	1.4			
BUN (mg/dL)	26.3	27.7	30.8	34.5	34.9	35.3	34.7
Creatinine (mg/dL)	2.46	3.99	5.13	4.92	4.65	3.33	1.91
eGFR (mL/min/1.73^2^)	27.66	15.83	11.83	12.42	13.27	19.5	37.04

On the following day (postoperative day 2), blood ketone levels were examined for fluctuating blood sugar levels, which disclosed a positive result (6.1 mmol/L) in favor of DKA. Regular insulin pump was titrated up to 2 U/h accordingly. Thereafter, the fever subsided and urine output increased gradually with concomitant recovery of blood pressure. Despite the occurrence of another episode of fever (38.3 °C) with significantly elevated procalcitonin (37.04 ng/mL) and C-reactive protein (291.41 mg/L) on postoperative day 5, in favor of a gout attack (blood uric acid 7.2 mg/dL), a non-enhanced abdominal computed tomography (CT) was still arranged in order to detect possible occult intra-abdominal infection or anastomotic leak. CT demonstrated neither fluid accumulation nor pneumoperitoneum. Therefore, the patient was immediately started with clear liquid diet and shifted to a full liquid diet on postoperative day 8 with the basal-bolus regular insulin regimen.

On postoperative day 10, we discontinued antibiotic use after the C-reactive protein and procalcitonin levels returned to the normal. His blood sugar remained stable after titration of insulin and oral hypoglycemic agent according to the endocrinologist’s suggestion. The patient was discharged on postoperative day 17. His renal function recovered gradually and no sequelae were noticed on 1-year follow-up.

High anion gap metabolic acidosis and ketonemia are the diagnostic adjuncts of DKA. While significant hyperglycemia (≥ 250 mg/dL) due to insulin deficiency remains the core etiology for this disease, mild hyperglycemia or even a euglycemic status could not completely exclude its clinical existence. DKA without marked hyperglycemia was defined as euglycemic DKA, which may manifest with subtle symptoms from dehydration and is usually induced by hyperglycemic status [1, 3].

Currently, one of the known risk factors for euglycemic DKA is the use of SGLT2 inhibitors. While the pathogenesis still remains unclear, excess urinary glucose excretion with relatively lower plasma glucose concentration may lead to an imbalance between insulin and glucagon (less insulin production from the beta cells, and further stimulation of the alpha cells). Moreover, the SGLT2 inhibitors can act independently on alpha cells with higher glucagon concentrations and eventually contribute to hepatic ketogenesis [3–8].

In an extensive review, most patients presenting with DKA after bariatric surgery were reported to have type 1 diabetes or were on treatment with SGLT2 inhibitors. This condition was extremely rare to be associated with T2DM morbidly obese patients or in the absence of SGLT2 inhibitor treatment [1]. General anesthesia, associated surgical stress, abrupt discontinuation of insulin, postoperative infection, prolonged starvation, poor oral intake, or severe dehydration may predispose DKA for those undergoing bariatric surgery [1, 7].

Moreover, DKA has been reported to usually present 1 to 6 weeks after the initial bariatric surgery among T2DM patients [2, 7]. Based on the similarity among manifestations of DKA and gastrointestinal symptoms due to bariatric surgeries, such as nausea or vomiting, timely identification and diagnosis may be difficult; nevertheless, our patient presented with euglycemic DKA sooner after initial RYGB (postoperative day 1), and the relative deficiency of insulin after this major surgery may be responsible for this condition. The recommendation of a basal insulin dosage of 0.3 U/kg of body weight during the perioperative stage as well as maintaining a target glucose level in the range of 140–180 mg/dL for the T2DM patients who will be undergoing bariatric surgery is essential [9]. In the early postoperative stage, diet modifications may be a key point for poor intake. Thus, dedicated titration of the insulin dose to avoid possible hypoglycemia is equally important [10].

Infection is another common predisposing factor for DKA, and procalcitonin can be a discriminatory biomarker for the detection of possible bacterial infection with concomitant leukocytosis and neutrophilia, which usually presents among those with DKA [2]. However, a previous case of a patient with DKA that had moderate elevation of procalcitonin without existing bacterial infection has been reported [11]. Furthermore, an acute attack of chronic gouty arthritis with febrile condition in our patient with concomitant euglycemic DKA could have been responsible for the relatively high procalcitonin level (37.04 ng/mL) [12, 13].

The rare occurrence of euglycemia DKA and complex manifestations of concurrent chronic kidney disease with acute exacerbation and a gout attack confused us during the first 3 days after initial RYGB. Though extremely rare, clinicians should not neglect the possibility of DKA for morbidly obese patients with T2DM after bariatric surgery, even with a euglycemic status and without a history of SGLT2 inhibitor treatment.
